# *Cis*-by-*Trans* Regulatory Divergence Causes the Asymmetric Lethal Effects of an Ancestral Hybrid Incompatibility Gene

**DOI:** 10.1371/journal.pgen.1002597

**Published:** 2012-03-22

**Authors:** Shamoni Maheshwari, Daniel A. Barbash

**Affiliations:** Department of Molecular Biology and Genetics, Cornell University, Ithaca, New York, United States of America; University of California Davis, United States of America

## Abstract

The Dobzhansky and Muller (D-M) model explains the evolution of hybrid incompatibility (HI) through the interaction between lineage-specific derived alleles at two or more loci. In agreement with the expectation that HI results from functional divergence, many protein-coding genes that contribute to incompatibilities between species show signatures of adaptive evolution, including *Lhr*, which encodes a heterochromatin protein whose amino acid sequence has diverged extensively between *Drosophila melanogaster* and *D. simulans* by natural selection. The lethality of *D. melanogaster*/*D. simulans* F1 hybrid sons is rescued by removing *D. simulans Lhr*, but not *D. melanogaster Lhr*, suggesting that the lethal effect results from adaptive evolution in the *D. simulans* lineage. It has been proposed that adaptive protein divergence in *Lhr* reflects antagonistic coevolution with species-specific heterochromatin sequences and that defects in LHR protein localization cause hybrid lethality. Here we present surprising results that are inconsistent with this coding-sequence-based model. Using *Lhr* transgenes expressed under native conditions, we find no evidence that LHR localization differs between *D. melanogaster* and *D. simulans*, nor do we find evidence that it mislocalizes in their interspecific hybrids. Rather, we demonstrate that *Lhr* orthologs are differentially expressed in the hybrid background, with the levels of *D. simulans Lhr* double that of *D. melanogaster Lhr*. We further show that this asymmetric expression is caused by *cis*-by-*trans* regulatory divergence of *Lhr*. Therefore, the non-equivalent hybrid lethal effects of *Lhr* orthologs can be explained by asymmetric expression of a molecular function that is shared by both orthologs and thus was presumably inherited from the ancestral allele of *Lhr*. We present a model whereby hybrid lethality occurs by the interaction between evolutionarily ancestral and derived alleles.

## Author Summary

When two different species mate, the hybrid progeny are often sterile or lethal. Such hybrid incompatibilities cause reproductive isolation between species and are an important mechanism for maintaining species as separate units. A gene called *Lethal hybrid rescue* (*Lhr*) is part of the cause of hybrid lethality between Drosophila species. Like many other hybrid incompatibility genes, *Lhr* protein sequences in the hybridizing species have diverged from one another by natural selection. This and other findings led to the hypotheses that the function of *Lhr* has changed between the two species, and this is what makes *Lhr* a hybrid lethality gene. Using a series of genetic, molecular, and cytological assays, we report evidence contrary to these hypotheses, that hybrid lethal activity is instead a function shared by both species and inherited from their common ancestor. This result is particularly surprising because the *Lhr* genes from the two species have different effects on hybrid viability. We discovered that these differential effects are caused by differences in expression levels of *Lhr* in hybrids rather than by changes in its protein-coding sequence. Our results demonstrate that, while natural selection may be important in evolving hybrid incompatibilities, how it does so in this case remains mysterious.

## Introduction

Species can be isolated from one another by a variety of reproductive barriers. One widely observed barrier is hybrid incompatibility (HI), the inviability or sterility of interspecies offspring. The key premise of the Dobzhansky-Muller (D-M) model explaining the evolution of HI is that genetic changes fixed in one population need not be compatible with changes fixed in a different population [1], [2]. This is most commonly illustrated as two independently evolving populations that each diverge from the ancestral state and fix new alleles. Hybridization between the two populations brings together the independently derived alleles, thereby generating a genotype unscreened by natural selection. This genotype may suffer from an incompatible interaction between the derived alleles, resulting in developmental breakdown of the hybrid progeny. A key feature of this model is that HI alleles have diverged in sequence and function (perhaps extensively) from their ancestral states. A second important prediction of the model is asymmetry: Gene “A” from species one may interact with gene “B” from species two to cause HI, but not vice-versa [3]. Questions fundamental to understanding speciation then are: What molecular divergence between the ancestral and derived alleles is causing HI? Is this divergence at the level of regulatory or structural changes? What are the evolutionary forces causing this divergence?

One unifying emerging trend is that HI loci often show high levels of divergence caused by natural selection [3], [4]. These findings are exciting, because if molecular divergence created by selection is causing HI, then the phenotypic target of selection is, at least in part, the evolutionary basis of speciation. A major goal then is to understand the role of selection in the evolution of incompatible divergence. Interestingly, studies on several recently characterized HI genes implicate divergence of heterochromatin and heterochromatin-binding proteins as the cause of incompatibility [5], [6]. As heterochromatin is the graveyard of selfish genetic elements, this functional divergence could be the legacy of genetic conflicts between the host species and the invasion of selfish DNAs such as transposable elements and satellite DNAs [7], [8].

A variation of the D-M model suggests that HI can also be caused by interactions between alleles that have not diverged from the ancestral state and derived alleles that have diverged in only one lineage [9]. If an HI allele has not diverged from its ancestral state, then this model predicts that its HI effects will be symmetrical, with orthologs from both species contributing to HI. Several examples of ancestral-derived incompatibilities have been discovered, and consistent with expectations the HI genes, when known, have experienced limited sequence divergence [10]–[12].

On the other hand, the expectation of a strict dichotomy between ancestral and derived HI alleles may reflect an over-simplified view of HI. Hybrids are the sum of two independently evolving genomes and thus suffer from multiple suboptimal interactions [3]. For example, species-specific divergence at *cis* and *trans*-regulatory elements is associated with widespread transcriptional dysregulation in hybrids [13], [14]. This creates a genetic background distinct from either parental species, and several well-studied HI genes have genetic properties in hybrids that are significantly different from or even opposite to their intraspecific roles [3].

Crosses between *D. melanogaster* females and *D. simulans* males produce inviable hybrid sons and sterile hybrid daughters [15]. The incompatible D-M interaction in hybrid males can in part be explained by the interaction between two genes, *Hybrid male rescue* (*Hmr*) on the *D. melanogaster* X-chromosome and *Lhr* on the *D. simulans* 2^nd^ chromosome [16]. A loss of function mutation in either *Hmr* or *Lhr* alone is sufficient to suppress the lethality of hybrid sons [16]–[19]. Thus it is the activity of these genes that causes hybrid breakdown.

*Lhr* (also known as *HP3*) encodes a protein that localizes to heterochromatin by directly binding to Heterochromatin Protein 1 (HP1) [16], [20], [21]. Population genetic analyses demonstrated that the *Lhr* protein coding sequence (CDS) has diverged extensively between *D. melanogaster* and *D. simulans* under positive selection, leading to the suggestion that *Lhr* has co-evolved with species-specific heterochromatin sequences [16]. If this co-evolution reflects a history of genetic conflict then one might predict that hybrid lethality is caused by defects in heterochromatin structure or maintenance, and that *Lhr* orthologs have functionally diverged in their heterochromatin localization properties such that they would mislocalize in the presence of heterochromatin from different species.

The hybrid lethality gene *Lhr* appeared initially to be a clear example of a derived D-M hybrid incompatibility locus. Consistent with the expectation of functional divergence, we previously found that the rescue of hybrid lethality via *Lhr* is asymmetric; removal of *D. simulans Lhr* (*sim-Lhr*) rescues lethal hybrid sons but removal of *D. melanogaster Lhr* (*mel-Lhr*) does not [16]. Surprisingly, however, *Lhr* orthologs from *D. melanogaster*, *D. simulans* and the outgroup species *D. yakuba* all have hybrid lethal activity when overexpressed in hybrids [21]. LHR proteins from these species also retain heterochromatic localization when expressed in polytenized salivary-gland cells, demonstrating that natural selection has not caused a wholesale change in *Lhr* function. This set of results suggests either that functional divergence is not an all-or-none property, or that *Lhr* is an ancestral HI locus, rather than a derived one.

To distinguish between these two possibilities, and to uncover the functional divergence underlying the asymmetric rescue properties of *Lhr* orthologs, we developed a native-promoter driven transgenic system that allows a sensitive comparison of the functions and localization properties of *D. simulans* and *D. melanogaster Lhr* orthologs. Using this system, we have compared *Lhr* function in both pure species and hybrids using three sets of experiments: (1) genetic tests for hybrid lethal activity and interaction with its D-M partner, *Hmr*; (2) detailed cytological mapping of the heterochromatic localization of LHR and its association with hybrid lethality, and (3) expression analysis comparing transcriptional levels of the *Lhr* orthologs.

## Results

### Both *D. simulans* and *D. melanogaster Lhr* have hybrid lethal activity under native expression conditions

**Figure 1:**
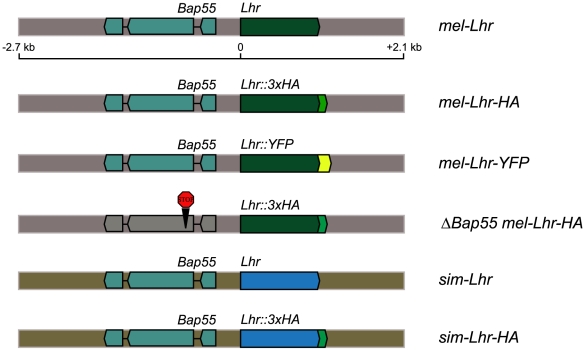
A schematic of the *Lhr* constructs. All constructs contain the full *Lhr* and *Bap55* coding sequences and UTRs. The “stop” in Δ*Bap55 mel-Lhr-HA* represents the insertion of two stop codons and a frame shift mutation. The *mel-Lhr* and *sim-Lhr* constructs are drawn to proportion, the HA and YFP epitope tags in other constructs are not drawn to proportion.

We generated parallel strains of *D. melanogaster* containing either *D. simulans Lhr* (*sim-Lhr*) or *D. melanogaster Lhr* (*mel-Lhr*) transgenes using the φC31 site-specific integration system [22]. Each *Lhr* ortholog was C-terminally tagged with an HA epitope and was expressed under the control of its native regulatory sequences (Figure 1). The transgenic constructs contained the eye-color marker *white^+^* and were each integrated into the *attP2* site on the third chromosome. We tested the transgenes for wild type activity by assaying for complementation of the *D. simulans Lhr^1^* hybrid rescue mutation. *D. simulans Lhr^1^* is a loss-of-function mutation that acts as a dominant suppressor of hybrid lethality [16], [18]. Complementation here means that the transgene provides sufficient wild type *Lhr* activity to suppress rescue by the *Lhr^1^* mutation, thus causing hybrid male inviability.

Complementation tests were performed by crossing *D. melanogaster* mothers heterozygous for an *Lhr-HA* transgene to *D. simulans Lhr^1^* fathers. This cross generates two classes of hybrid sons: the control class that lacks the transgene and has white eyes, and the experimental class that inherits the transgene and has orange eyes. Complementation is detected as the lethality of orange-eyed sons. If hybrid lethal activity partitions discretely between *Lhr* orthologs, as expected from the functional divergence interpretation of genetic asymmetry, sons inheriting the φ{Dsim\Lhr-HA} transgene should be lethal, while those inheriting φ{Dmel\Lhr-HA} should be viable.

Unexpectedly, both transgenes fully complemented the *D. simulans Lhr^1^* mutation (Table 1, crosses 1 thru 4), suggesting that both *D. simulans* and *D. melanogaster Lhr* orthologs have hybrid lethal activity. As this result was contrary to expectation we tested several possible causes of artifacts. First, the C-terminal HA-tag does not affect *Lhr* function because untagged versions of both *mel-Lhr* and *sim-Lhr* also complement *Lhr^1^* (Table 1, crosses 5 and 6). Second, the adjacent gene *Bap55* present in these constructs is not responsible for complementation because a modified *mel-Lhr-HA* transgene, φ{ΔBap55 Dmel\ Lhr-HA}, in which the *Bap55* CDS is interrupted by two stop codons and a frameshift mutation, also complements *Lhr^1^* (Table 1, cross 7). Third, the results are not caused by other unknown aspects of the strain background or by the *attP2* site because the *attP2* site itself without an integrated transgene does not complement *Lhr^1^* (Table 1, cross 8). Furthermore *mel-Lhr-HA* integrated into a different site (*attP86Fb*) also complements *Lhr^1^* (Table 1, cross 4). Fourth, these results are not due to an over-expression artifact because data presented below demonstrate that the *mel-Lhr-HA* transgene expresses *Lhr* at a level similar to the endogenous wild type locus (see section “*cis*-by-*trans* regulatory divergence causes functional divergence of *D. melanogaster* and *D. simulans Lhr*” below). These results clearly show that *D. melanogaster Lhr* has hybrid lethal activity even when expressed at its wild type level.

**Table 1:** *D. melanogaster* and *D. simulans Lhr* orthologs suppress hybrid rescue by *D. simulans Lhr*.*^1^*

					No. of hybrid males	
Cross	Transgenic construct	*attP* integration site	Temp.	No. of hybrid females	Genotype 1 +/+	Genotype 2 *φ{ }/+* (or *attP2/+*)
1	*φ{sim-Lhr-HA}*	*attP2*	RT	232	92	0
			18°C	214	110	0
2	*φ{sim-Lhr-HA}*	*86Fb*	RT	135	74	0
			18°C	100	57	0
3	*φ{mel-Lhr-HA}*	*attP2*	RT	177	91	0
			18°C	240	122	0
4	*φ{mel-Lhr-HA}*	*86Fb*	RT	263	121	0
			18°C	246	109	0
5	*φ{sim-Lhr}*	*attP2*	RT	184	61	0
6	*φ{mel-Lhr}*	*attP2*	RT	302	150	0
			18°C	217	84	0
7	*φ{ΔBap55, mel-Lhr-HA}*	*attP2*	RT	324	156	0
			18°C	322	188	0
8	none	*attP2*	RT	280	NA	160

Crosses were between *D. melanogaster* females heterozygous for the different transgenes tested (*w; ϕ{transgene, w^+^}*) and *D. simulans Lhr^1^* males. Transgenes are denoted as *φ{}* in the table. The transgenes carry a copy of the *w^+^* gene so the hybrid sons inheriting the transgene (genotype 2) were distinguished from their control siblings (genotype 1) by their eye-colour, except for cross 8 where *D. melanogaster* females homozygous for the integration site without an inserted transgene were mated to *D. simulans Lhr^1^* males. NA = not applicable. RT = room temperature.

How can these results be reconciled with the original observation that only a mutation in *D. simulans Lhr*, and not the *D. melanogaster* ortholog, rescues hybrid sons? Those experiments were done in hybrid genotypes that had only a single dose of either *mel-Lhr* or *sim-Lhr* [16]. In contrast, the experiments here were performed by adding a transgenic copy of either *mel-Lhr* or *sim-Lhr* to hybrids that also carried the endogenous chromosomal copy of *mel-Lhr*. Increased dosage of *mel-Lhr* in the current experiments may therefore explain why we have not observed a difference between the *mel-Lhr* and *sim-Lhr* transgenes. This hypothesis raises the question of whether the hybrid lethal activity of the *mel-Lhr-HA* transgene would be eliminated in a background lacking the chromosomal copy of *mel-Lhr*. To test this we crossed *D. melanogaster* mothers that were doubly heterozygous for the *mel-Lhr-HA* transgene and an *Lhr^−^* deficiency to *D. simulans Lhr^1^* fathers. If transgenic *mel-Lhr* behaves identically to the endogenous locus, then hybrid sons inheriting the *Lhr^−^* deficiency along with the *mel-Lhr* transgene should be equivalent in *Lhr* dosage to rescued *+/Lhr^1^* hybrid males and thus be viable. However, hybrid sons from this cross were also inviable (Table S3). This result indicates that the *mel-Lhr-HA* transgene does not precisely phenocopy the native chromosomal *mel-Lhr* locus. In the Discussion we consider possible causes of this difference.

### Interactions with *Hmr* reveal a difference in lethal activity of *Lhr* orthologs

Because the complementation tests did not reveal a difference in the hybrid lethal effects of *Lhr* orthologs we used a more sensitive genetic assay to test for functional divergence between *mel-Lhr* and *sim-Lhr*. We previously demonstrated that *Lhr*-dependent hybrid lethality requires the presence of its D-M partner, the *D. melanogaster* gene *Hmr* [16].

We reasoned that the hypomorphic allele *Hmr^1^* might exhibit different sensitivities to the HI effects of the different *Lhr* alleles, but that the null allele *Df(1)Hmr^−^* would not. We therefore introduced each of our *Lhr* transgenes into these *Hmr* mutant backgrounds and tested the effect of the transgenes on hybrid male viability in crosses to *D. mauritiana* and *D. simulans*. Crosses with the *sim-Lhr-HA* transgene recapitulated our previous experiments: *Hmr^1^* hybrid males carrying *sim-Lhr-HA* were essentially inviable at room temperature and showed strongly reduced viability at 18°C, while *Df(1)Hmr^−^* hybrid males were equally viable with and without the transgene (Table 2). We then performed similar crosses with *mel-Lhr-HA*. This transgene had little effect on viability of males with the null mutation *Df(1)Hmr^−^* and the results were in general not significantly different compared to the crosses with *sim-Lhr-HA* (Table 2, sets 1 & 2). In crosses with the hypomorphic mutation *Hmr^1^*, hybrids carrying *mel-Lhr-HA* had reduced viability compared to their non-transgene carrying siblings, particularly at room temperature. Strikingly, we found that in all four cross conditions the magnitude of the viability reduction was significantly less for *mel-Lhr-HA* compared to *sim-Lhr-HA* (Table 2, sets 3 & 4). These data demonstrate that *sim-Lhr* is more potent than *mel-Lhr* in creating the hybrid lethal interaction with *Hmr*, and that our *Lhr* transgenes thus do in fact reveal a significant degree of functional divergence.

**Table 2:** *D. simulans Lhr* interacts more strongly with *Hmr* than *D. melanogaster Lhr*.

				No. of hybrid sons with transgene tested				
				*mel-Lhr*		*sim-Lhr*		
Set	*Hmr* allele tested	Male parent	Temp	*+/+*	*Φ{}/+*	*+/+*	*Φ{}/+*	Fisher's exact test P
1	*Df(1)Hmr^−^*	*D. sim*	RT	128	160	278	288	0.21796
	*Df(1)Hmr^−^*	*D. sim*	18°C	n.d.	n.d.	29	18	n.d.
2	*Df(1)Hmr^−^*	*D. mau*	RT	124	195	119	124	0.02024*
	*Df(1)Hmr^−^*	*D. mau*	18°C	140	120	50	45	0.904438
3	*Hmr^1^*	*D. sim*	RT	181	33	35	0	0.00654**
	*Hmr^1^*	*D. sim*	18°C	349	258	502	82	0.00000***
4	*Hmr^1^*	*D. mau*	RT	351	117	159	2	0. 00000***
	*Hmr^1^*	*D. mau*	18°C	497	388	476	256	0.00029***

Transgenic *Lhr* orthologs were tested for interaction with two *Hmr* alleles: a null and a hypomorph, specified in the table as *Df(1)Hmr^−^* and *Hmr^1^* respectively. *D. melanogaster* female parent genotypes were: null mutation, *y w Df(1)Hmr^−^ v/FM6*; *ϕ{transgene, w^+^}/+* and hypomorph, *w Hmr^1^ v*; *ϕ{transgene, w^+^}/+*. Transgenes are denoted as *φ{}* in the table. Each genotype was mated separately to males from *D. simulans v* or *D. mauritiana Iso105*. Hybrid male progeny that inherit the transgene are orange eyed, while the sibling brothers are white eyed. The FET is comparing the relative viability of hybrid sons inheriting the *D. melanogaster Lhr* transgene *vs* the relative viability of sons inheriting the *D. simulans Lhr* transgene in parallel crosses. (n.d. = not determined;

*, *p*≤0.05;

**, *p*≤0.01;

***, *p*≤0.001). RT = room temperature.

### A sensitive genetic screen reveals weak hybrid rescue by deletion of *mel-Lhr*

Having demonstrated that wild type *mel-Lhr* has hybrid lethal activity, we reinvestigated whether removal of *mel-Lhr* has any detectable hybrid rescue activity. We previously showed that deletion of *mel-Lhr* does not rescue hybrids with *D. simulans* [16]. We therefore looked for rescue in hybrids with *D. mauritiana* at 18°C, conditions that are maximally conducive for hybrid viability [17]. Unrescued hybrid males die as larvae [23]. We found that two *D. melanogaster Lhr* ^−^ deletions rescued 7–21% of males to the pharate adult stage (Table 3). This is clearly a modest rescuing effect and did not occur in one of the genetic backgrounds tested (*Df(2R)BSC49* crossed to *D. mauritiana* W139), but it is significant because crosses with 45 other deletions across chromosome 2R gave no rescue. A third *Lhr* ^−^ deletion, *Df(2R)BSC44*, did not rescue hybrids, demonstrating that hybrid viability is sensitive to genetic background effects. The difference in magnitude of rescue for deletion of *mel-Lhr* versus *sim-Lhr* further supports our conclusion using transgenes that *sim-Lhr* has greater hybrid lethality activity than *mel-Lhr*.

**Table 3:** Rescue of *D. melanogaster/D. mauritiana* hybrid male lethality by *D. melanogaster Lhr^−^* deletions.

		No. of F1 hybrid females		No. of F1 hybrid males			
Deletion tested	*D. mauritiana* male parent	*Df/+*	*Balancer/+*	*Df/+*	*Balancer/+*	Pharate adult	Estimated pharate male viability
*l(2)k01209*	Iso 105	54	35	0	0	4	7.4%
	W139	291	305	0	1	24	8.2%
*Df(2R)BSC49*	Iso 105	70	63	0	0	15	21.4%
	W139	142	35	0–3^a^	0–3^a^	0	0
*Df(2R)BSC44*	*w f*	47	44	0	0	0	0
	Iso 105	87	85	0	0	0	0
	W139	60	80	0	0	0	0
Other	various	6643	6589	12	8	0	0

*D. melanogaster* females with the designated deletions were mated to *D. mauritiana* males at 18°. The small number of live males recovered are likely patroclinous exceptions, that is *X^mau^/O*. Pharate adults were scored after all adults had eclosed. “Estimated pharate male viability” was calculated as number of pharate adult males/number of *Df/+* females. For crosses with *l(2)k01209*, pharate adult males displayed the orange-eye color characteristic of *Df/+*. For crosses with *Df(2R)BSC49*, the genotypes of the pharate adults could not be determined and were thus assumed to be *Df/+*. Full genotypes of *Lhr^−^* deletions are: *y^1^,w^67c23^*; *P{w^+mC^ = lacW}l(2)k01209[k08901a]/CyO*; *Df(2R)BSC49/SM6a*; and *Df(2R)BSC44/SM6a*. All three were previously shown to be deleted for *Lhr* [16]. “Other” refers to 45 different *Lhr^+^* deletions spanning chromosome arm 2R.

a Cross produced 3 total males, which were not genotyped for Df or balancer.

### LHR partially localizes to the dodeca satellite within heterochromatin in *D. melanogaster*

**Figure 2:**
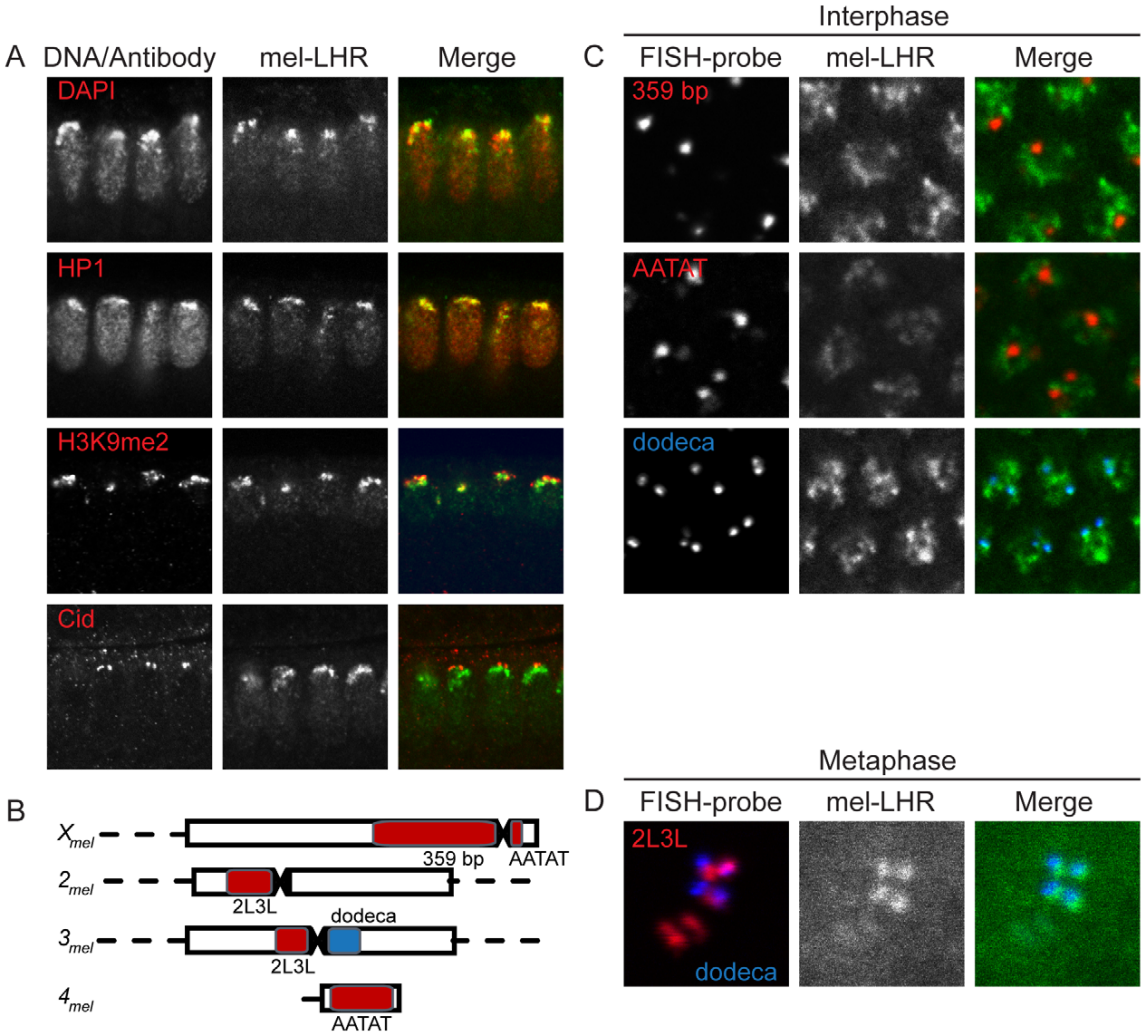
Localization of *D. melanogaster* LHR within pericentric heterochromatin. (A) *D. melanogaster* cycle 14 embryos co-stained for mel-LHR-HA (green) and different heterochromatic markers (red). mel-LHR-HA localizes as distinct foci within heterochromatin marked by DAPI and anti-HP1, and shows colocalization with pericentric heterochromatin marked by anti-H3K9me2. mel-LHR-HA does not colocalize with centromeres as marked by anti-Cid. (B) Schematic of satellites used as targets of FISH. Note that AATAT is also present on the Y chromosome [47]. (C) Immuno-FISH experiments in *D. melanogaster* embryos with anti-HA (green) to detect mel-LHR-HA and various FISH probes (red or blue). In interphase nuclei LHR shows no overlap with the 359 bp and AATAT satellites but partially colocalizes with the dodeca satellite. (D) A mitotic nucleus with the pericentric regions of chromosomes 2 and 3 (marked by the 2L3L satellite) aligned at the metaphase plate. In the merge LHR signal is clearly present only at the 3rd chromosome, marked by the dodeca satellite.

We next set out to determine why *sim-Lhr* is more potent than *mel-Lhr* in causing hybrid lethality. Coding sequence evolution leading to different protein localization patterns is one possible cause of *Lhr* functional divergence. In order to test this hypothesis we examined the cellular localization of LHR orthologs in their wild type backgrounds using our *Lhr* transgenes. In *D. melanogaster* LHR protein is most abundant during embryogenesis (Figure S1). We therefore analyzed the distribution of LHR during early embryogenesis and found a cyclical on-off pattern through the cell cycle, with localization to chromatin mainly during interphase (Figure S2). This pattern is identical to its interaction partner, Heterochromatin Protein 1 (HP1) [24]. Thus, we focused on interphase nuclei, and unless otherwise specified all images were taken at embryonic nuclear cycles 12–14, when heterochromatin is first observed. Consistent with previous results, LHR-HA colocalized with HP1 at DAPI-rich heterochromatic foci on the apical surface of the nuclei (Figure 2A). Unlike HP1, however, which is found throughout the nuclear compartment including euchromatin, LHR is restricted to heterochromatin. Consistent with being localized to a sub-domain of HP1, LHR strongly overlapped with Histone-3 lysine 9 dimethylation (H3K9me2), a histone modification specific to pericentric heterochromatin [25], but not with Cid, a histone variant specific to the centromere.

LHR was also observed in the embryonic germline precursors, the pole cells, and in the somatic and germline cells of the ovary (Figure S3A), where it again colocalized with H3K9me2 (Figure S3B). However, LHR was excluded from the nucleolus, a sub-compartment within heterochromatin consisting of rDNA repeats (Figure S3B). This observation suggested that LHR has a specific distribution within heterochromatin. We therefore used immuno-FISH to investigate the localization pattern of LHR relative to various pericentric satellites in *D. melanogaster*. We observed no overlap between LHR and the 359 bp satellite, a 4–5 Mb block on the X-chromosome [26], [27], nor between LHR and the highly abundant AATAT satellite, which is distributed across multiple chromosomes [28] (Figure 2C). In contrast, LHR consistently overlapped with dodeca, a G/C-rich pericentric satellite on the third chromosome [29], although a substantial amount of LHR is also found in other heterochromatic regions that we have not mapped. During metaphase, however, four discrete foci of LHR were visible along the metaphase plate. Noticeably, each LHR focus corresponded to the pericentric region of the third chromosome, as identified by overlapping dodeca signal (Figure 2D).

### sim-LHR also associates with the dodeca satellite in *D. simulans*

**Figure 3:**
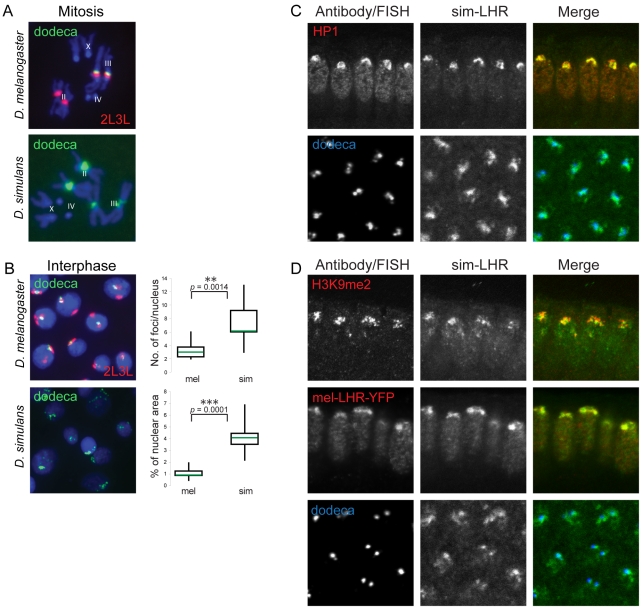
LHR orthologs have conserved localization properties despite species-specific divergence of heterochromatin. (A,B) The dodeca satellite has diverged in its chromosomal location and interphase organization between *D. melanogaster* and *D. simulans*. FISH to mitotic (A) and interphase (B) nuclei from 3^rd^ instar larval brain cells with probes to dodeca (green) and 2L3L (red). Right panels in part B show quantification of the nuclear distribution of the interphase dodeca FISH signals. The mean values are indicated by the green lines (n = 10 for each sample).Boxes span the interquartile range and whiskers extend to the maximum and minimum values. Significance was tested by Wilcoxon rank-sum test. mel = *D. melanogaster*; sim = *D. simulans*. (C, D) Conserved heterochromatic localization properties of LHR orthologs. (C) *sim-Lhr-HA* transgene in *D. simulans* embryos. Top panel, Anti-HA (green) detects sim-LHR-HA colocalizing with HP1 (red) in the apical heterochromatin. Bottom panel, sim-LHR-HA (green) partially colocalizes with dodeca satellite (blue). (D) sim-LHR-HA (green) expressed in *D. melanogaster* embryos colocalizes with H3K9me2 (red) and with mel-LHR-YFP, detected with anti-GFP (red). sim-LHR-HA also partially overlaps with the *D. melanogaster* dodeca satellite (blue).

We next tested whether LHR localization is conserved in *D. simulans*. We constructed transgenic lines of *D. simulans* using the sim-Lhr-HA construct described above. Like mel-LHR in *D. melanogaster*, sim-LHR in *D. simulans* also localized to apical heterochromatic foci, as marked by DAPI (Figure 3C). We were particularly interested to determine whether sim-LHR associated with the dodeca satellite, because the distribution of dodeca varies among *melanogaster* subgroup species [30]. In particular, dodeca satellite is present only in the pericentric region of the third chromosome in *D. melanogaster*, but is present in the pericentric heterochromatin of both the second and the third chromosomes in *D. simulans* [30]. We confirmed this difference and found that the dominant dodeca signal is on the *D. simulans* second chromosome in mitotic brain squashes (Figure 3A). We also noted significant differences in the interphase organization of dodeca between species. We quantified the number of dodeca foci per nucleus and the fraction of nuclear space occupied in interphase nuclei from wild type brains. The dodeca signal in *D. simulans* appeared fragmented into more foci and occupied a greater nuclear volume, indicating that dodeca-containing heterochromatin has evolved species-specific nuclear organization properties (Figure 3B).

Despite this divergence in both chromosomal location and structure of dodeca, immuno-FISH mapping in *D. simulans* showed that sim-LHR partially colocalized with dodeca in interphase nuclei (Figure 3C). As with mel-LHR, a substantial amount of sim-LHR localizes to other regions of heterochromatin which we have not mapped. However, our results show that its association with dodeca is conserved between species.

We were unable to detect sim-LHR on chromosomes during metaphase (data not shown). We note, however, that only a small fraction of mel-LHR appears to be on metaphase chromosomes in *D. melanogaster* (see Figure S2) and we have found that challenging to image. We are thus unable to determine whether the apparent absence of sim-LHR from metaphase chromosomes reflects a true difference between species or instead is due to technical limitations.

### sim-LHR colocalizes with mel-LHR within *D. melanogaster*

It is unclear how LHR localizes to specific domains within heterochromatin, but it might require associations with other heterochromatin proteins, some of which are also rapidly evolving [21]. If LHR is co-evolving with other rapidly evolving proteins, then its heterochromatic localization might be altered when expressed in a foreign species.

To test this possibility we examined the localization of sim-LHR-HA in *D. melanogaster*. We found that sim-LHR-HA localized to the H3K9me2-enriched heterochromatic regions (Figure 3D), and colocalized with the dodeca satellite in a pattern identical to that seen for mel-LHR above (see Figure 2C). In order to directly compare the localization of LHR orthologs within the same nucleus, we generated a recombinant transgenic line that expressed both YFP-tagged mel-LHR and HA-tagged sim-LHR. The two LHR orthologs showed complete overlap, demonstrating that the heterochromatic localization properties of LHR orthologs are conserved (Figure 3D).

### Incompatible hybrids have wild-type heterochromatin states and LHR localization

**Figure 4:**
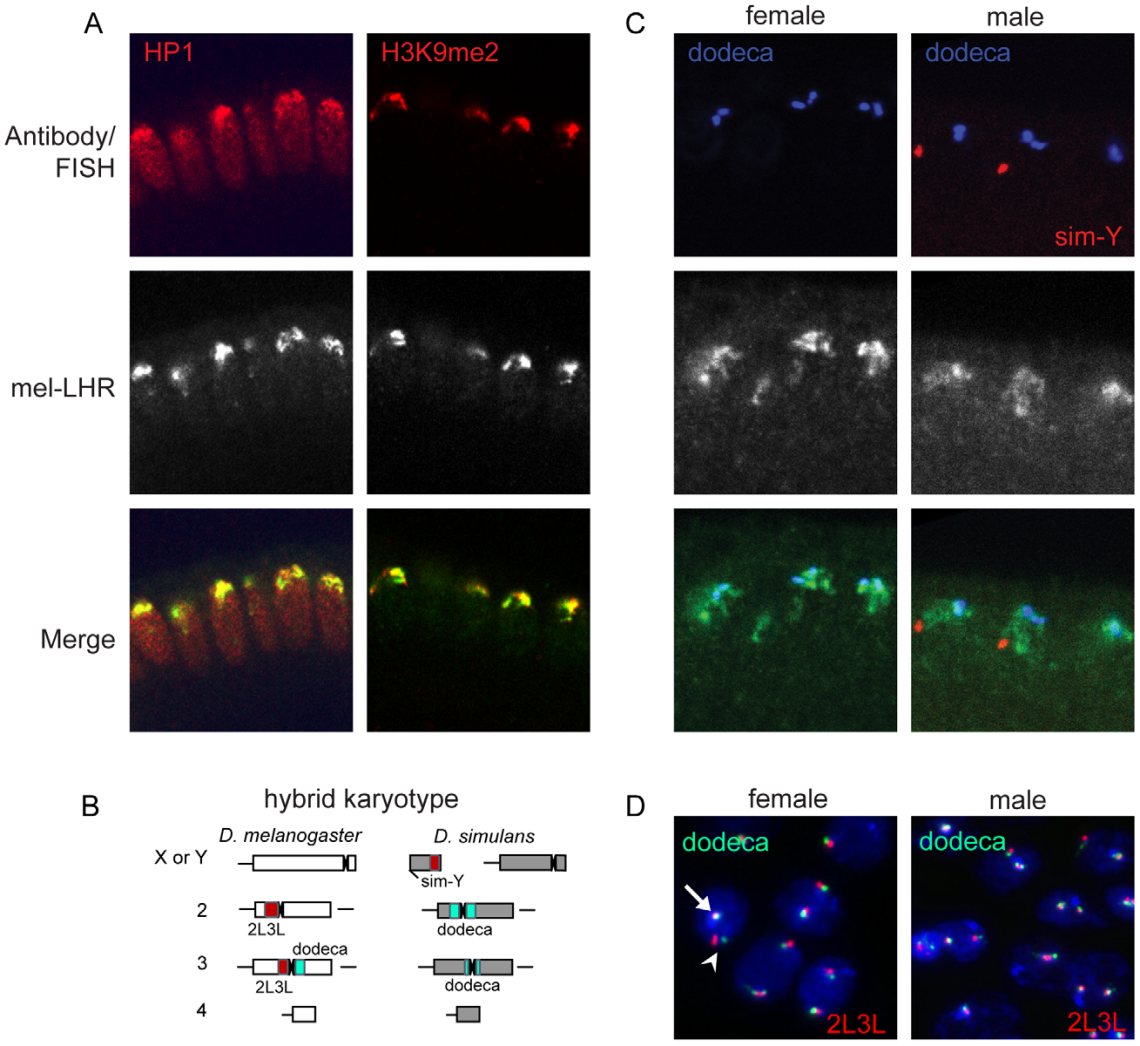
Normal LHR localization and organization of heterochromatin in hybrids. (A) mel-LHR-HA (green) colocalizes with HP1 and H3K9me2 (each red), similarly to wild type (see Figure 2). (B) A schematic karyotype of a hybrid nucleus with sites of FISH probe hybridization highlighted. Red = 2L3L, blue = dodeca. (C) mel-LHR-HA (green) partially colocalizes to dodeca satellite (blue) in male and female hybrid embryos. (D) Interphase nuclei from brain cells of male and female larvae have wild type organization of the dodeca and 2L3L satellites (see Figure 3B for *D. melanogaster* wild type control). The orthologous second chromosomes are identifiable as a pair of adjacent red and green signals (arrowhead), while the *D. melanogaster* third chromosome is visible as the overlapping red and green signal (arrow). Hybrid larvae were generated from a cross between *D. melanogaster yv* females and *D. simulans v* males, and were sexed using mouth hook coloration (males are *y* in phenotype and females are *y^+^*).

To determine whether heterochromatin states are perturbed in hybrids we examined HP1 and H3K9me2 localization. Although hybrid embryos were not sexed in this experiment, the staining appeared uniformly wild type in all embryos (Figure 4A). In order to specifically compare LHR and/or dodeca localization in hybrid males versus females, we developed a FISH probe that hybridized to the *D. simulans* Y-chromosome (Figure S4). We found that mel-LHR staining was enriched within apical heterochromatin in both sexes, and that it overlapped partially with dodeca (Figure 4C). Importantly, we detected no difference in dodeca organization and LHR localization between lethal hybrid males and viable hybrid females. Since heterochromatin defects might become more apparent later in development we then looked at heterochromatin states in hybrid larval neuroblasts. Consistent with the embryo staining, we saw no defects in the organization of either dodeca or the 2L3L satellite in either inviable male or viable female larvae (Figure 4D). Furthermore, despite differences in the pericentric heterochromatic sequences between homologous chromosomes, somatic pairing during interphase appeared unaffected in hybrid nuclei.

### *cis*-by-*trans* regulatory divergence causes functional divergence of *D. melanogaster* and *D. simulans Lhr*

**Figure 5:**
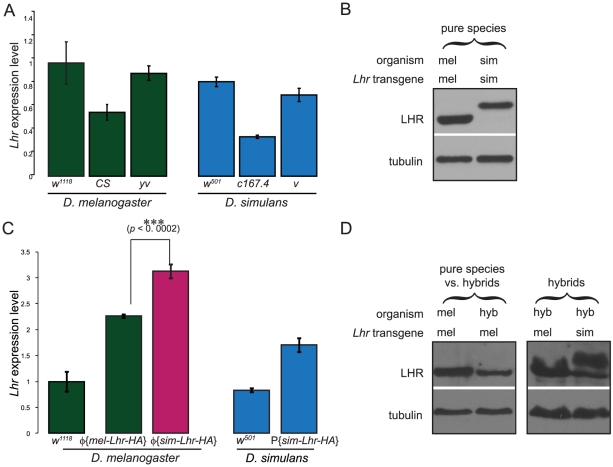
*Cis*-by-*trans* regulatory divergence of *D. simulans Lhr*. (A) *Lhr* transcript levels in different wild type and marker strains of *D. melanogaster* and *D. simulans* (nested ANOVA F_1;4_ = 0.89; *p* = 0.39 for between species variation). (B) LHR protein levels are similar between *D. melanogaster* (mel) and *D. simulans* (sim). Western blot detecting HA-tagged LHR transgenes expressing in their own species. sim-LHR-HA migrates higher than mel-LHR-HA. (C) *Lhr* transcript levels in transgenic lines compared to the corresponding host strain genetic background (*w^1118^* for *D. melanogaster* and *w^501^* for *D. simulans*). The transgenic lines are homozygous for the transgene and for the endogenous *Lhr* allele, and therefore have four copies of *Lhr*. *Lhr* transcript abundance is approximately doubled in φ*{mel-Lhr-HA}* and *P{sim-Lhr-HA}* relative to their respective reference backgrounds, indicating wild type expression levels of these transgenes in their native species. In contrast, *Lhr* transcription in φ*{sim-Lhr-HA}* is significantly higher than in φ*{mel-Lhr-HA}* (by two-tailed *t*-test) and is ∼3× the level of the reference background. (D) LHR protein levels are not increased in hybrids. Left, one copy of the *mel-Lhr-HA* transgene in *D. melanogaster* and in hybrids; right, one copy of the *mel-Lhr-HA* or *sim-Lhr-HA* transgenes in hybrids. The origin of the lower band in the *sim-Lhr-HA* lane in hybrids is unclear. For A and C, RNA was isolated from 6–10 hr old embryos. *Lhr* expression levels were measured relative to *rpl32* using quantitative RT-PCR. Expression levels were normalized by setting the *D. melanogaster w^1118^* strain to 1. Error bars represent standard deviation within biological replicates, n≥6 for all except *P{sim-Lhr-HA}* where n = 3. For B and D, protein extracts were from 0–16 hr embryos and the immunoblots were hybridized with anti-HA antibodies. Anti-tubulin antibodies were used as a loading control.

In spite of the adaptive protein sequence divergence between *D. melanogaster* and *D. simulans* orthologs of *Lhr*, our results surprisingly suggest only a limited degree of functional divergence of *Lhr*, with both orthologs having significant hybrid lethal activity and similar patterns of protein localization within heterochromatin. We therefore asked if gene regulatory divergence of *Lhr* between *D. melanogaster* and *D. simulans* might instead be responsible for the asymmetry of the lethal effects of *Lhr* in hybrids. We first surveyed *Lhr* transcript levels using qRT-PCR in three strains from each of the two species, and found no significant difference between the two species (Figure 5A). Consistent with this, we detected similar levels of LHR protein between the species (Figure 5B). Expression levels of *mel-Lhr-HA* and *sim-Lhr-HA* transgenes were each at a wild type level in their own species background, as total *Lhr* transcript level was approximately double in strains homozygous for the transgenes compared to wild type controls (Figure 5C). However, *sim-Lhr* was significantly overexpressed in *D. melanogaster*. The different expression levels of the *sim-Lhr-HA* and *mel-Lhr-HA* transgenes in the same *D. melanogaster* background indicate that *cis*-regulatory divergence has occurred at *Lhr* (Figure 5C). Furthermore, the fact that wild type levels of *Lhr* are not significantly different between the species (Figure 5A) despite these *cis*-regulatory differences suggests that *trans* acting factors that regulate *Lhr* have diverged. Taken together these data demonstrate that *Lhr* has undergone *cis*-by-*trans* compensatory regulation, such that *cis*-regulatory regions and *trans*-factors have co-evolved within each species to maintain a constant level of gene expression [31]. The uncoupling of such species-specific compensatory changes in a foreign genetic background would explain why *sim-Lhr* is hyper-expressed in *D. melanogaster*.

**Figure 6:**
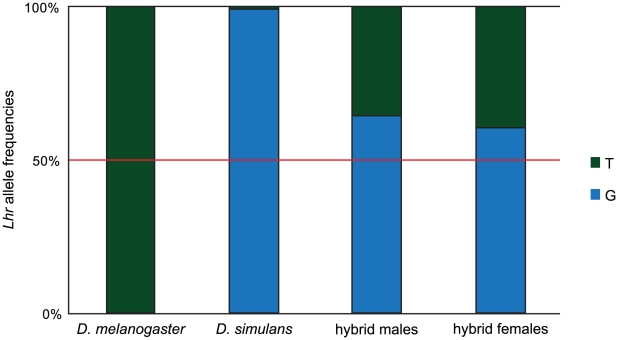
Asymmetric expression of *Lhr* orthologs in hybrids. Pyrosequencing across a SNP fixed between *Lhr* orthologs was used to measure the ratio of allelic transcription in pure-species and hybrid larvae that were 3–5 days old. *Lhr* transcript from pure species *D. melanogaster* is 100% for the *D. melanogaster*-specific variant of the SNP. For *D. simulans* 2 out of 3 of the technical replicates from one of the cDNA preparations showed 100% of transcripts with the *D. simulans*-specific variant of the SNP; a small amount of the *D. melanogaster*-specific SNP detected in the 3rd replicate most likely reflects error or contamination. The *D. simulans*-specific SNP is detected at levels significantly greater than the expected 50% in both male and female hybrids (*p*<.0001 and *p* = .005 for hybrid males and females, respectively, by two-tailed *t*-test).

Given these results, we hypothesized that such a mechanism might cause asymmetric expression of *Lhr* orthologs in hybrids and by extension underlie the asymmetric rescue properties of *Lhr* orthologs. To test this hypothesis, we did allele-specific pyrosequencing to estimate the relative expression levels of the two *Lhr* orthologs in hybrids (Figure 6). We examined 3–5 day-old larvae because temperature shift experiments have shown that the L2/L3 stage is the critical phase of the lethality [17]. As expected *Lhr* transcript from the pure species parents was essentially 100% for their respective species-specific SNP. However, there was a significant overrepresentation of the *D. simulans*-specific SNP in both hybrid males and females, with ∼65% of *Lhr* transcripts deriving from the *D. simulans* ortholog in hybrid males and ∼60% in hybrid females. These data confirm our expectation that *cis*-by-*trans* divergence of *Lhr* regulation causes asymmetric expression in hybrids, and strongly suggests that a *D. simulans* mutation rescues hybrid sons because it removes a greater fraction of the total pool of *Lhr*, compared to a mutation in the *D. melanogaster* ortholog. We emphasize that this regulatory evolution leads to asymmetric expression of *Lhr* in hybrids but does not appear to cause an increase in total levels. The abundance of transgenic mel-LHR protein is not elevated in hybrids compared to pure species, as determined by Western blots (Figure 5D). Moreover, because protein levels of LHR orthologs appear equivalent in hybrids, we infer that levels of *D. simulans* LHR are also not visibly elevated in hybrids (Figure 5D). We therefore conclude that hybrid male lethality is not caused by *Lhr* over-expression. As we discuss below, lethality instead appears to result from hybrids becoming sensitive to *Lhr* activity due to its interaction with additional genes including *Hmr*.

## Discussion

*Lhr* and *Hmr* are D-M interaction partners that cause hybrid lethality [16]. Population genetic analyses of *Lhr*, *Hmr* and other HI genes found their coding sequences to be evolving rapidly under positive selection [3], [4]. These results imply that selection-driven protein divergence is the molecular basis of incompatibility in hybrids. An experimental prediction then is that independently evolving orthologs of a D-M gene should be non-equivalent with respect to the HI phenotype. Our initial genetic data supported this expectation for *Lhr*, because a loss of function mutation in *D. simulans Lhr* rescues lethal hybrid sons, while a loss-of-function mutation in *D. melanogaster Lhr* does not [16]. These findings led to several hypotheses: 1) HI is due to divergence specific to the D. *simulans* lineage; 2) this divergence has caused significant changes in the heterochromatin association properties of LHR proteins from *D. melanogaster* and *D. simulans*; and 3) defects in heterochromatin states directly cause hybrid lethality.

Contrary to some of these expectations, a subsequent study found that both *D. melanogaster* and *D. simulans Lhr* could cause HI when overexpressed in hybrids and that both proteins localized to heterochromatin when ectopically expressed in salivary gland cells [21]. In order to further explore functional differences between *sim-Lhr* and *mel-Lhr* we developed a native-promoter-driven transgenic system and performed higher resolution mapping of LHR protein localization. We found that both *Lhr* orthologs suppress hybrid rescue by *D. simulans Lhr^1^*, supporting the inference that hybrid lethal activity is a shared ancestral function. However, using a more sensitive interaction assay with *Hmr*, we detected that the lethal interaction was greater with *sim-Lhr* (Table 2). This finding is consistent with the pattern of genetic asymmetry where a mutation in *D. simulans Lhr* rescues hybrid lethality, while a deficiency removing *D. melanogaster Lhr* does not [16]. Our further investigation here reveals that removing *mel-Lhr* does in fact provide a modest level of hybrid rescue (Table 3). The fact that this rescue only occurs to the pharate adult stage in a minority of male hybrids underscores our conclusion that *mel-Lhr* has weaker hybrid lethal activity than *sim-Lhr*. A major focus of this study then became to understand the cause of this difference.

### Assessing transgene function

We attempted to create transgenic constructs of *Lhr* that were functionally identical to the wild type locus. To achieve this we generated *Lhr* transgenes that were driven by their native *cis*-regulatory sequences (Figure 1). Although the boundary of the regulatory regions included in the constructs was arbitrary we did quantitative RT-PCR assays on the transgenes to confirm that they expressed at wild type levels in both *D. melanogaster* and *D. simulans* (Figure 5C). Additionally, we infer from western blots that the abundance of transgenic LHR protein is similar in hybrids and pure species (Figure 5D), suggesting comparable expression levels in both backgrounds.

Nevertheless, we found that our *mel-Lhr-HA* transgene has greater activity than wild type *Lhr* when directly tested against an *Lhr^−^* deletion (Table S3). We consider two explanations: One possibility is that the construct has aberrant expression in a limited number of tissues or developmental stages that is beyond the resolution of detection in qRT-PCR assays of whole embryos or animals. Two, genetic assays for *Lhr* rescue are highly sensitive to genetic background effects; for example a large screen for suppression of *Lhr* rescue found a wide range of rescue even in the control balancer-chromosome classes [32]. We also observed here variable effects of *D. melanogaster Lhr^−^* deletions on hybrid viability (Table 3). Thus it is possible that this anomalous result results from an interaction with the multi-locus deficiency used and/or its genetic background.

While the result in Table S3 remains unexplained, we emphasize that the major conclusions of this study are not affected. The inference that *mel-Lhr* has hybrid lethal activity is independently shown by the rescue activity of the *mel-Lhr* deletion (Table 3). That result also demonstrates the asymmetric lethal activity of *mel-Lhr* and *sim-Lhr*, as does pyrosequencing of cDNA from hybrids (Figure 6). Likewise, the inference from transgenic assays that *Lhr* has undergone *cis*-by-*trans* compensatory evolution (Figure 5C) is fully consistent with the quantification of *Lhr* transcription by qRT-PCR in pure species (Figure 5A) coupled with the pyrosequencing result in hybrids.

### Conserved heterochromatic localization of LHR orthologs

Our first hypothesis to explain the differential effects of *mel-Lhr* versus *sim-Lhr* on hybrid viability was that their respective proteins might have different localization patterns. Previous studies found the LHR localizes to heterochromatin in *D. melanogaster*, but did not determine whether it is a general heterochromatin factor or instead has a specific localization within heterochromatin [16], [20], [21]. The heterochromatic landscape is dramatically different in closely related species [33], which raises the question of whether rapid evolution of *Lhr* orthologs reflects functional divergence necessitated by its association with fast-evolving heterochromatic sequences.

We addressed this question by (1) mapping LHR localization within *D. melanogaster* pericentric heterochromatin, (2) comparing its localization in *D. simulans*, and (3) examining sim-LHR localization in a *D. melanogaster* background. Within both species LHR localized to heterochromatic foci but was not ubiquitous (Figure 2A). For example, mel-LHR does not overlap with the AATAT or the 359 bp satellites, two major components of *D. melanogaster* pericentric heterochromatin [28]. In contrast, a portion of LHR consistently colocalized with the dodeca satellite in both species during interphase. The conservation of this colocalization pattern was particularly striking, given that dodeca repeats are found only on chromosome III in *D. melanogaster* but on both chromosomes II and III in *D. simulans* (see Figure 3A and reference [30]). Thus, the chromosomal distribution of LHR between the two species is different.

However, despite this divergence in the genomic location of dodeca, sim-LHR when expressed in *D. melanogaster* colocalized perfectly with mel-LHR (Figure 3D), demonstrating full conservation of LHR's heterochromatic localization properties. For three reasons, it is highly unlikely that this conserved pattern is because LHR orthologs share a DNA-binding activity specific to the dodeca sequence. First, LHR contains no recognizable DNA-binding domain. Second, LHR localization to heterochromatin is dependent on HP1 binding [20], [21]. Finally, LHR signal is neither restricted to dodeca nor perfectly overlapping with it (Figure 2C and Figure 3C). Thus, it is unclear what features of DNA or chromatin are configuring this localization pattern of LHR.

### No evidence for heterochromatic defects or satellite DNA-mediated genetic conflicts in incompatible hybrids

Neither the structure of the dodeca satellite nor LHR localization differed between pure species and hybrids, nor between lethal male and viable female hybrids (Figure 4C and 4D). These results set *Lhr* apart from two other well-characterized heterochromatin-associated HI genes. OdsH is a fast-evolving homeodomain protein that mislocalizes to the heterochromatic Y-chromosome in hybrids [5]. *Zhr* is a species-specific satellite DNA that causes hybrid lethality by improperly segregating during mitosis [6]. Such defects have been interpreted as support for the hypothesis that internal conflict with selfish heterochromatic elements is driving HI [3], [4], [7], [8]. We cannot rule out the possibility that there are defects in heterochromatin undetectable by our cytological analyses, or that *Lhr* may have other functions related to telomeric [16] or euchromatic [20] localization that have been affected by genetic conflicts. Nevertheless, the observations that heterochromatin appears normal in hybrids and that LHR localizes normally in both hybrids and when expressed in foreign species are not consistent with straightforward expectations of genetic conflict theories involving satellite DNAs [3]. Further work will be required to understand how *Lhr* causes lethal hybrids to have defects in cell proliferation and abnormally few larval cells entering mitosis [34], [35].

### Regulatory divergence causes the asymmetric hybrid lethal effects of *Lhr* orthologs

Having found that LHR orthologs have not diverged in their heterochromatin localization, we tested whether the asymmetric effects of mutations in *mel-Lhr* versus *sim-Lhr* on hybrid lethality reflect a history of regulatory sequence divergence rather than protein sequence divergence. In particular, we hypothesized that asymmetric expression of *Lhr* orthologs in hybrids could explain the aforementioned genetic asymmetry. We tested this hypothesis by measuring allele-specific expression of *Lhr* orthologs in hybrid larvae. Our results strongly support this hypothesis: we found that approximately 66% of the total *Lhr* transcripts in lethal hybrid male larvae originates from the *D. simulans* allele (Figure 6). Thus a mutation in *D. simulans Lhr* creates hybrid sons with only 1/3^rd^ the wild type level of *Lhr* transcript, while hybrid sons with a mutation in the *D. melanogaster* ortholog have twice that amount. We conclude that only a loss-of-function mutation in *D. simulans Lhr* produces viable hybrids because it removes a greater proportion of the total *Lhr* gene product.

The divergence leading to asymmetric expression does not, however, reflect species-specific divergence in expression levels, because *Lhr* expression is not significantly different between *D. melanogaster* and *D. simulans* (Figure 5A). Instead asymmetric *Lhr* expression in hybrids is likely caused by the uncoupling of species-specific compensatory changes between *cis*-regulatory sequences and *trans*-factors. Interestingly, studies comparing the evolution of transcriptional networks between species have found that this type of regulatory divergence is frequently associated with gene mis-expression in interspecific hybrids [14], [31]. Furthermore, Takahasi et al. recently found evidence that stabilization of expression levels within a species involves widespread *cis*- and *trans*-compensatory mutations that can be detected as incompatibilities between heterospecific regulatory elements in interspecific hybrids [36]. The authors also suggest that signatures of adaptive evolution might result from the rapid accumulation of compensatory changes, and thus reflect the maintenance of an existing function rather than the evolution of a novel one. To our knowledge *Lhr* is the first example of *cis*-by-*trans* compensatory evolution occurring at an adaptively evolving hybrid incompatibility gene. An intriguing possibility is that the rapid evolution of the protein coding region reflects compensatory changes required to maintain an existing regulatory function of *Lhr*, rather than to alter its protein function.

### A Dobzhansky-Muller interaction between a derived and an ancestral allele

We emphasize that *cis*-by-*trans* regulatory divergence explains the asymmetric effect of *Lhr* mutations on hybrid viability, but is not the direct cause of *Lhr* having hybrid lethal activity. Instead our data argue that the hybrid male genotype has evolved an acute sensitivity to *Lhr* dosage. Our genetic assays further suggest that the activity of *Lhr* that causes hybrid lethality was likely present in the ancestral state because it is shared by both *mel-Lhr* and *sim-Lhr*. This hypothesis is further supported by the observation that GAL4-UAS driven expression of *Lhr* from *D. yakuba*, an outgroup species, also kills hybrid sons [21]. Unlike *Lhr*, however, transgenic assays with its D-M partner, *Hmr*, showed that only the *D. melanogaster* ortholog but not the *D. simulans* ortholog is capable of causing hybrid lethality [37]. That result is consistent with the HI effect of *Hmr* being derived during evolution in the *D. melanogaster* lineage.

**Figure 7:**
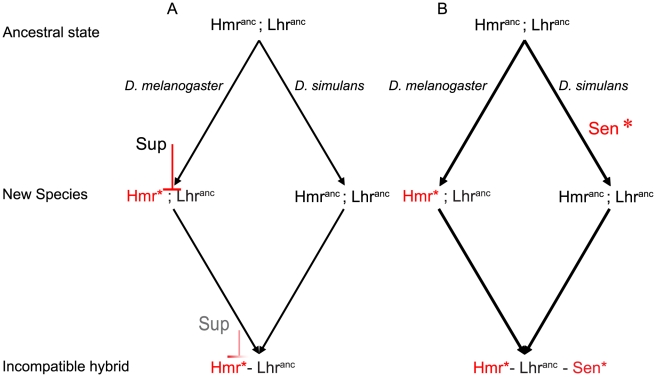
Alternative models for the evolution of hybrid lethality: incompatibility between an evolutionarily derived *D. melanogaster Hmr* and ancestral *Lhr*. In the first model a suppressor (*Sup*) fixed in the *D. melanogaster* lineage prevents the two-locus D-M interaction in the pure-species background, but is inactivated or suppressed in the hybrid background. In the second model an additional sensitizing locus (Sen*) from the *D. simulans* lineage is needed to complete the lethal interaction. Additional sensitizing loci could exist in both lineages, leading to a complex multi-genic interaction. The models depicted could involve either direct or indirect physical interactions among genes and gene products.

HI involving ancestral gene function is compatible with the D-M model, and was first considered by Muller [9]. One model he proposed involves incompatibility between an ancestral and a derived allele, with loss of a suppressor allele being required to ‘release’ the incompatibility. Here, this would require a suppressor to evolve first and become fixed in the *D. melanogaster* lineage, before the incompatibility-causing substitutions evolved in *Hmr* (Figure 7A). In the hybrid background, the suppressor is diluted or inactivated, exposing the lethal interaction. Alternatively, incompatibility could result from a complex epistatic interaction involving three or more loci. In the simplest case, changes at a single *D. simulans* locus, Sen*, cause the hybrid background to become sensitive to the dosage of *Lhr* in the presence of *Hmr* from the *D. melanogaster* lineage (Figure 7B). We favor the latter model because in the first model over-expression of *sim-Lhr* in *D. melanogaster* might be expected to at least partially overcome the suppressor and create the incompatible interaction. However, GAL4-UAS over-expression of *sim-Lhr* has no effect in a *D. melanogaster* pure species background [16], [21].

Although we diagram only a single sensitizing locus, a polygenic model involving multiple genes is equally possible, because available data only establish that *Hmr* and *Lhr* are insufficient to cause hybrid lethality [16]. If many additional genes are involved, then the distinction between ancestral and derived alleles may become blurred. For example, interacting genes may co-evolve, and have high evolutionary rates that maintain interactions rather than alter molecular functions.

Other examples of ancestral-derived incompatibilities have been discovered, such as the inter-allelic incompatibility at the *S5* locus in rice, and the bi-locus incompatibility between the derived *S. cerevisiae* splicing factor *MRS1* and the ancestral *COX1* mRNA [11], [12]. However, unlike the incompatible *S5* alleles which differ by only two amino acid substitutions, and *COX1* which retains the ancestral intron that causes HI, *Lhr* orthologs have diverged rapidly under selection [16]. It is therefore remarkable that despite extensive protein sequence divergence between the hybridizing species, hybrid lethality has evolved as sensitivity to the dosage of an ancestral function. The key mechanistic implication is that instead of searching for a process or function that differentiates *Lhr* orthologs as the source of hybrid lethality, we now know that the sensitivity to *Lhr* in hybrids is based on a function and/or interaction that is common to both orthologs.

### Role of positive selection in the evolution of hybrid incompatibilities

There are least 6 HI genes known that are rapidly diverging under selection [3]. With the exception of *OdsH* and *Prdm9*, where the signature of selection is restricted to a single functional domain [38], [39], in the other HI genes peaks of nonsynonymous substitutions do not coincide with a specific functional domain within the protein coding sequence. In these cases, it has been assumed that changes derived under selection have led to functional divergence, in turn causing incompatibility. However, it remains to be tested if that is truly the case.

We have assayed the hybrid lethal activity of both *Lhr* orthologs and found that despite extensive selection-driven divergence of the protein sequence, hybrid lethal activity is a shared ancestral function. We do not rule out the possibility that protein divergence makes some minor difference in hybrid lethal activity. However, our results suggest that the asymmetric effect of *Lhr* in causing hybrid lethality is explained by regulatory divergence. This finding demonstrates the need to consider regulatory divergence when interpreting interspecies experiments. Our results also highlight the complexity of the interspecific background and emphasize that hybrids are far from being the stoichiometric sum of two parental genomes. We suggest that while positive selection of protein-coding sequences remains a characteristic of HI genes, the phenotypic target of selection and its connection to HI are in some cases much less direct than expected.

## Materials and Methods

### Drosophila crosses and stocks

All crosses were done at room temperature, or at 18°C where explicitly stated. At least 2 replicates were done for each cross. Each interspecific cross was initiated with ∼15–20 1-day-old *D. melanogaster* virgin females and ∼30–40 3–4-day-old sibling-species males. The nomenclature used for the transgenic lines and a complete description of the constructs used to generate them are included in Table S1. Genetic markers, deficiencies, and balancer chromosomes are described on FlyBase [40]. We previously showed that the *D. melanogaster* stock *y^1^,w^67c23^*; *P{w^+mC^ = lacW}l(2)k01209[k08901a]/CyO*, used here in Table 3 and Table S3, is deleted for *Lhr* (see Fig. S4 in ref. [16]).

### DNA constructs

To make a modified pCasper4 containing the attB site, we PCR amplified a 280 bp fragment using the pTA plasmid (gift from Michele Calos) as the template [22]. This PCR product, along with flanking *Sal*I sites was cloned into the compatible *Xho*I site of pCasper4 to create the plasmid pCasper4\attB. In order to construct *Lhr* transgenes with *Lhr* under the control of its native regulatory sequences, we used a 4.8 kb genomic fragment that spans 2.7 kb upstream and 1 kb downstream of the *Lhr* CDS. This fragment includes the complete CDS of the adjacent gene *Bap55* (Figure 1).

To generate the p{sim-Lhr} construct we amplified this fragment from *D. simulans w^501^* genomic DNA, using primer pairs 691/664 (see Table S2 for primer sequences). This PCR product was gel purified and cloned into the pCR-BluntII TOPO vector (Invitrogen), according to manufacturer's directions. The insert was sequenced completely and subcloned into pCasper4\attB using *Not*I and *Kpn*I restriction enzymes. Note that this transgene contains more upstream DNA than the *sim-Lhr* transgene used by Prigent et. al. [41], which was also functional.

The p{mel-Lhr} construct was generated similarly, a 4.8 kb fragment was PCR amplified from wild type *D. melanogaster* (strain Canton-S) genomic DNA using primer pairs 597/598, and TOPO cloned into pCR-BluntII vector. The forward primer contains a *Not*I site, allowing the insert to be released as a *Not*I fragment and cloned into the *Not*I site of pCasper4\attB. A clone was chosen with the same orientation as in p{sim-Lhr}.

To construct p{sim-Lhr-HA} a triple-HA tag was added in-frame to the C-terminus of the *Lhr* CDS using a two-piece fusion PCR strategy. The two overlapping PCR products were amplified using p{sim-Lhr} as the template, with primer pairs 691/728 and 729/664. These fragments were used as templates for the fusion PCR, and the gel-purified product was TOPO cloned into the pCRBluntII vector and sequenced completely. The insert was then subcloned into pCasper4\attB exactly as in p{sim-Lhr}. The construction of p{mel-Lhr-HA} followed the same logic, using the primer pairs 597/728 and 729/598. To synthesize the p{mel-Lhr-YFP} construct a three-piece fusion PCR strategy was used, the first and last PCR products, containing upstream and downstream genomic regions respectively, were amplified using p{mel-Lhr} as the template, with primer pairs 597/730 and 733/598. The central PCR product containing the YFP-tag was amplified from p{*w^+mC^* UAS-Lhr::Venus = UAS-Lhr::YFP} [16], with primer pair 731/732. The 3 overlapping PCR products were used as templates for the fusion PCR, and cloned into the pCR-BluntII vector and sequenced completely. The insert was subcloned into pCasper4\attB exactly as in p{mel-Lhr}.

The p{ΔBap55 mel-Lhr-HA} construct is identical to p{mel-Lhr-HA} except that the *Bap55* CDS is interrupted by the insertion of “TAA TGA C”, i.e. two stop codons and a frame shift mutation after the second methionine at position 6. Two overlapping PCR products were amplified using p{mel-Lhr-HA} as template, with primer pairs 597/1171 and 1172/598. The products were stitched together using fusion PCR and cloned into pCasper4\attB exactly as done in p{mel-Lhr}.

### Transgenic fly lines

*φ*C31-mediated transformation of *D. melanogaster* was performed by Genetic Services Inc. The integration sites used were: i) *P{CaryP}attP2* and ii) *M{3xP3-RFP.attP}ZH-86Fb* at cytological positions 68A4 and 86Fb, respectively [22], [42]. *P{CaryP}attP2* carries the body color marker *yellow^+^* (*y^+^*). Site specificity of integration was tested using the PCR assays of ref. [43]. We also developed attP docking-site specific PCR assays, primer pairs1086/1087 for attP2, and 949/1177 for ZH-86Fb. All *D. melanogaster* transformants were crossed into the strain *w^1118^*. P-element mediated integration was used to transform the *D. simulans w^501^* strain with P{sim-Lhr-HA}.

### Quantitative RT–PCR

Total RNA was isolated using the Trizol Reagent (Invitrogen), followed by DNaseI (Roche) treatment and purification using RNeasy columns (Qiagen). First strand cDNA was synthesized from 4 µg of total RNA using the SuperScriptIII first-strand synthesis system (Invitrogen) with the oligo(dT)_20_ primer in a 20 µl reaction according to the manufacturer's instructions. Quantitative real time PCR (qRT-PCR) was performed on a Biorad MyiQ cycler with SYBR detection using the 2× supermix from Biorad. Relative concentrations of *Lhr* transcripts were calculated against *rpl32* as the reference gene with *rpl32* primers from reference [44]. The *rpl32* gene sequence is 99% identical between the species. For *Lhr* primer pair 1147/1148 was developed to recognize conserved sequences and to amplify both *D. melanogaster* and *D. simulans Lhr* with equal and high efficiency. For each sample real-time PCR on test and reference genes was done in technical triplicates, and the standard curve method was used to estimate transcript abundance. For each genotype RNA was isolated from between 3 and 4 independent 6–10 hr-old embryo collections. For all genotypes except *D. simulans* P{sim-Lhr-HA} cDNA was synthesized twice from each RNA isolate.

### Pyrosequencing

RNA was extracted from 3–5 day-old larvae collected from non-crowded vials. In hybrid crosses the *D. melanogaster* mothers carried the X-linked mutation *y^−^* allowing the sex of larvae to be determined by using mouth hook coloration (daughters are *y^+^* and sons *y^−^*). Total RNA and genomic DNA were simultaneously extracted from the same biological samples using the SV RNA system (Promega). For the pure species control, RNA and genomic DNA were extracted once from a single biological collection, followed by a single round of cDNA synthesis. For the hybrid samples, RNA and genomic DNA were extracted from four independent biological samples. cDNA was synthesized twice from each independent RNA isolate. Pyrosequencing measurements were performed in triplicate on each cDNA and in duplicate on each genomic DNA.

### Western blotting

Whole cell extracts were obtained by grinding samples in ∼3 volumes of lysis buffer (50 mM Tris-HCl pH 7.5, 10 mM EDTA, 1.25% TritonX-100, 1× Roche protease inhibitor tablet). Extracts were cleared by centrifugation at 14,000 rpm for 10 min at 4°C. Total protein concentration of the cleared extracts was measured using Bradford assay (Biorad) and the samples were boiled in 0.5× volume of 4× SDS-Sample buffer. For most westerns 40 µg of total protein was loaded in each lane. Primary antibodies used were: rat anti-HA 3F10 (Roche; 1∶1000) and mouse anti-tubulin T5168 (Sigma; 1∶10,000). HRP conjugated goat anti-rat and goat anti-mouse secondary antibodies (Jackson; 1∶5,000) were used and detected with ECL Western blotting substrate (Pierce).

### FISH and immuno-staining

Embryo FISH and immuno-FISH were performed as in reference [6] and immunostaining of ovarioles was performed as in reference [45] with the following antibodies: Rat anti-HA 3F10 (Roche; 1∶100), mouse anti-HP1 C1A9 (DSHB; 1∶100), rabbit anti-histone H3 lysine 9 dimethylation (Upstate 07-441; 1∶100), rabbit anti-Cid (a gift from S. Henikoff; 1∶1000), rabbit anti-GFP (Abcam ab6556; 1∶300), mouse anti-Fibrillarin (Cytoskeleton Inc. AFb01; 1∶400) and mouse anti-Hts 1B1 (DSHB; 1∶4). FISH probes are described in reference [6]. DNA was stained using TOPRO-3 iodide (Molecular Probes) or Vectashield containing DAPI (Vector Laboratories). All imaging was conducted at the Cornell University Core Life Sciences Microscopy and Imaging Facility, using either a Leica DM IRB confocal microscope or an Olympus BX50 epifluorescent microscope, except for embryo images with a DAPI channel which were taken in the Plant Cell Imaging Center at the Boyce Thompson Institute, with a Leica TCS SP5 confocal microscope. Images were processed using Photoshop (Adobe, version 7.0). Contrast and brightness changes, when used, were applied globally across images.

Quantification of dodeca signal in interphase larval brain tissue was done using ImageJ [46]. Watershed segmentation was applied on the DAPI-channel to generate a mask of nuclear territories. The Analyze Particle function was then used to identify individual nuclei as ROIs (regions of interest) and screened to exclude aberrant nuclear segmentations and non-nuclear entities. Each ROI was individually selected on the dodeca FISH channel of the same image and the FociPicker3D plug-in was used to identify regions of local maxima. We then calculated two measures to estimate the nuclear dispersion of dodeca satellite: (1) the total number of foci per nucleus and (2) the fraction of total nuclear area occupied by the dodeca signal.

## Supporting Information

Figure S1 Comparison of LHR protein across developmental stages using Western blots. A developmental time course of mel-LHR-HA protein from a transgene homozygous in *D. melanogaster*. In a longer exposures LHR is present in all stages, including adult females, however relative levels are highest during embryogenesis. This is consistent with *Lhr* transcript profiles published in ref. [48].
(EPS)

Figure S2 LHR distribution through the cell cycle. mel-LHR-HA detected with anti-HA (green) and DNA stained with TOPRO-3 (red) in *D. melanogaster* nuclear cycle 10 embryos.
(TIF)

Figure S3 LHR localizes to heterochromatin in germline cells. (A) Left, the posterior region of a cycle 12–14 embryo showing mel-LHR-HA (green) in the pole cells (germline precursor cells; arrow). Right, a germarium from a 2–3 day old female ovary with the anterior end to the left; mel-LHR-HA is found in all germline cells including the stem cells (arrow) and the developing cysts (arrowhead). The fusome and follicle cell membranes are marked using anti-hts (red). A portion of an egg chamber is visible in the bottom right. (B) Stage 4–6 egg chambers from 2–3 day-old *D. melanogaster* females, stained for mel-LHR-HA (green) and heterochromatic markers (red). mel-LHR-HA is found in both the polyploid germline nurse cells (large cells in the centre) and in somatic follicle cells (small surrounding cells), and colocalizes to a sub-compartment of heterochromatin marked by anti-H3K9me2 but not to the nucleolus, stained with anti-fibrillarin.
(EPS)

Figure S4 FISH mapping identifying (AATAAAC)n as a *D. simulans* Y-specific satellite. (AATAAAC)n and (AATAC)n were identified as satellite sequences on the *D. melanogaster* Y by Bonaccorsi & Lohe [47]. The left panel contains a *D. melanogaster* female and male embryo. Both (AATAC)n and (AATAAAC)n hybridize specifically to the male embryo. The inset is a higher magnification of nuclei from the male embryo. (AATAC)n does not hybridize to *D. simulans* (data not shown). The right panel shows that (AATAAAC)n hybridizes to a single region on the Y-chromosome in a mitotic spread from wild type *D. simulans* male 3^rd^ instar larval brain cells. The inset is nuclei from a *D. simulans* embryo, presumed to be male; (AATAAAC)n signal is seen as a single dot in each nucleus.
(EPS)

Table S1 Transgene nomenclature.
(DOCX)

Table S2 Primers used.
(DOCX)

Table S3 A single dose of transgenic *mel-Lhr* suppresses hybrid rescue by *D. simulans Lhr^1^*. *D. melanogaster* females of the genotype *y^1^,w^67c23^*; *P{w^+mC^ = lacW}l(2)k01209[k08901a]/CyO*; φ*{mel-Lhr-HA}/+* were mated to *D. simulans Lhr^1^* males. *P{w^+mC^ = lacW}l(2)k01209[k08901a]* is abbreviated as *Df(Lhr)* and is described in Materials and Methods. Hybrid males were scored as follows: *Df(Lhr)/Lhr^1^* progeny are *Cy^+^* and have straight wings; *Bal/Lhr^1^* progeny carry the *CyO* balancer chromosome and have *Cy* (curly) wings. The *attP2* site into which φ*{mel-Lhr-HA}* is integrated is marked with the *yellow^+^* body-color gene. Hybrid male progeny that inherit the transgene are therefore wild type for body color, while the sibling brothers are yellow bodied. Hybrid female progeny with and without the transgene cannot be distinguished because they inherit the X chromosome from *D. simulans Lhr^1^* fathers that is wild type for the *yellow* locus. RT = room temperature.
(DOCX)
