# Supplementary figures and images for: Cis-by-Trans Regulatory Divergence Causes the Asymmetric Lethal Effects of an Ancestral Hybrid Incompatibility Gene
Source: PLoS Genet. 2012 Mar 22;8(3):e1002597. doi: 10.1371/journal.pgen.1002597 (PMC3310770; doi:10.1371/journal.pgen.1002597)

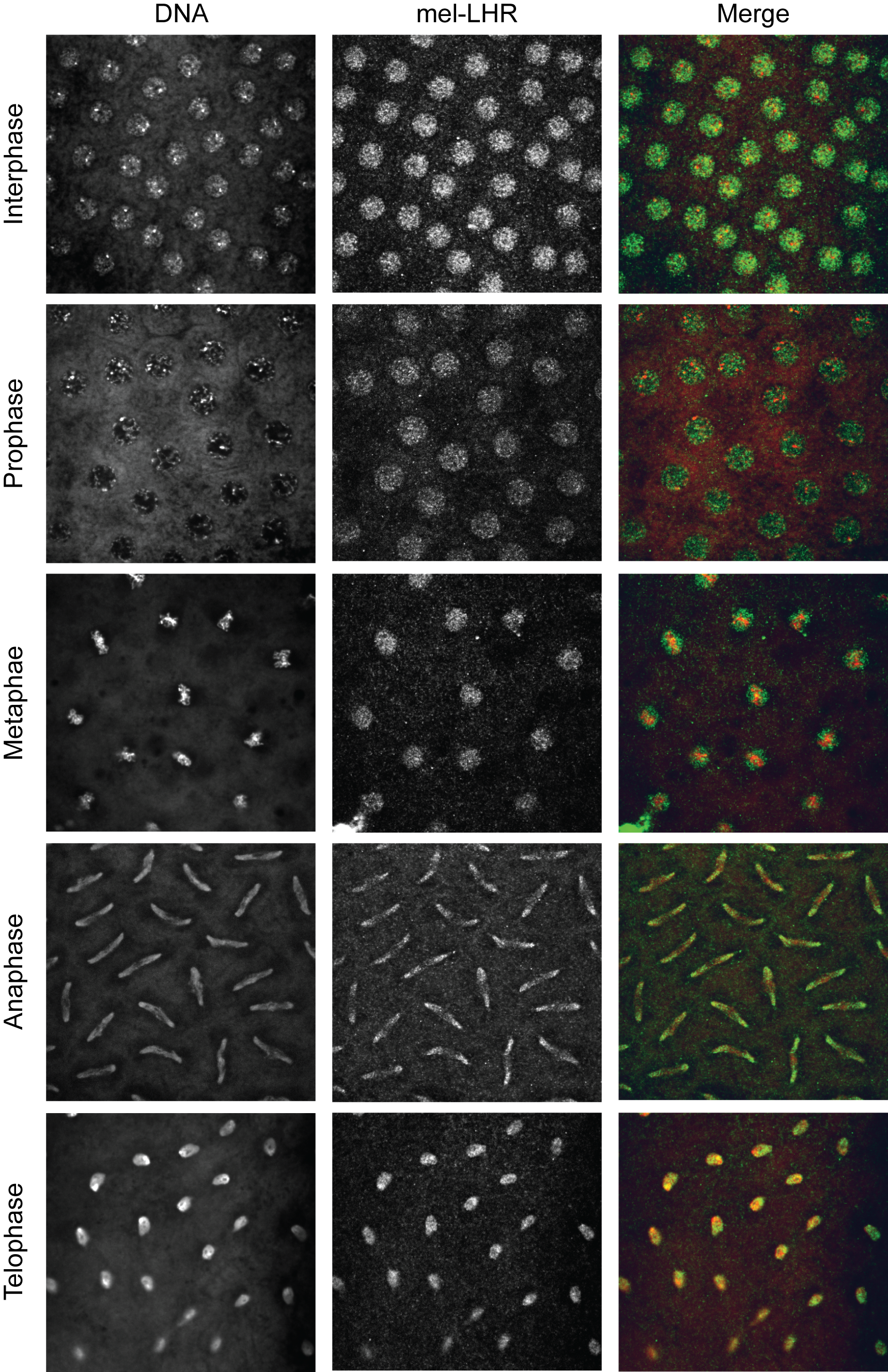

Supplement: Figure S2 — LHR distribution through the cell cycle. mel-LHR-HA detected with anti-HA (green) and DNA stained with TOPRO-3 (red) in D. melanogaster nuclear cycle 10 embryos. (TIF) [file pgen.1002597.s002.tif]
