# Supplementary material for: Cis-by-Trans Regulatory Divergence Causes the Asymmetric Lethal Effects of an Ancestral Hybrid Incompatibility Gene
Source: PLoS Genet. 2012 Mar 22;8(3):e1002597. doi: 10.1371/journal.pgen.1002597 (PMC3310770; doi:10.1371/journal.pgen.1002597)
Supplement: Table S1 — Transgene nomenclature. (DOCX) [file pgen.1002597.s005.docx]

Table S1: Transgene nomenclature

| No. | Full name of construct | Transgene short-hand |
| --- | --- | --- |
| 1 | *P{w^+mC^ Bap55^t4.8^ Lhr^t4.8^*$=$ *mel-Lhr}* | *Φ{mel-LHR}* |
| 2 | *P{w^+mC^ Dsim\Bap55^t4.8^ Dsim\Lhr^t4.8^*$=$ *sim-Lhr}* | *Φ{sim-LHR}* |
| 3 | *P{w^+mC^ Bap55^t4.8^ Lhr::HA^t4.8^*$=$ *mel-Lhr-HA}* | *Φ{mel-LHR-HA}* |
| 4 | *P{w^+mC^ Bap55^t4.8^ Lhr::YFP^t4.8^*$=$ *mel-Lhr-YFP}* | *Φ{mel-LHR-YFP}* |
| 5 | *P{w^+mC^ Dsim\Bap55^t4.8^ Dsim\Lhr::HA^t4.8^*$=$ *sim-Lhr-HA}* | *Φ{sim-LHR-HA}* |
| 6 | *P{w^+mC^ Bap55^͞^ Lhr::HA^t4.8^*$=$ *ΔBap55 mel-Lhr-HA}* | *Φ{ΔBap55 mel-LHR-HA}* |
