# Supplementary material for: Cis-by-Trans Regulatory Divergence Causes the Asymmetric Lethal Effects of an Ancestral Hybrid Incompatibility Gene
Source: PLoS Genet. 2012 Mar 22;8(3):e1002597. doi: 10.1371/journal.pgen.1002597 (PMC3310770; doi:10.1371/journal.pgen.1002597)
Supplement: Table S2 — Primers used. (DOCX) [file pgen.1002597.s006.docx]

Table S2: Primers used.

| **No.** | **Sequence** | **Capitalized region** |
| --- | --- | --- |
| 691 | tactatAAGCTTtggttgttccacacgactttatcg | HindIII |
| 664 | tcgcatAAGCTTctggcaggtggtaaccgatacgg | HindIII |
| 597 | gggtttGCGGCCGCttccacacgactttatcgacagga | NotI |
| 598 | gggtttGGATCCcggctcctcaaacattcctttatg | BamHI |
| 728 | TGCATAGTCCGGGACGTCATAGGGATAGCCCGCATAGTCAGGAACATCGTATGGGTACATtgttctcagcgtaggccg | 3xHA tag |
| 729 | CCCTATGACGTCCCGGACTATGCAGGATCCTATCCATATGACGTTCCAGATTACGCTtgactttctttcgtataaaatgc | 3xHA tag |
| 730 | tgttctcagcgtaggccgccttgagc |  |
| 731 | TCAAGGCGGCCTACGCTGAGAACAaagg | YFP tag |
| 732 | atgtgcattttatacgaaagaaagTCACGTGGACCGGTGCTTGTACAGC | YFP tag |
| 733 | TACAAGCACCGGTCCACGTGActttctttcgtataaaatgcacataag | YFP tag |
| 1171 | gcaccatgTAATGACctatatggcggcgacgagat | Stop codon |
| 1172 | ccatatagGTCATTAcatggtgccgccactcat | Stop codon |
| 1086 | gtcgcccacatgacacaag |  |
| 1087 | ctctttgcaaggcattacatctg |  |
| 949 | gtcgacgatgtaggtcacggtc |  |
| 1177 | gcagccgaacgaaattaaaa |  |
| 1147 | gcatttagaagtggaggtcctcg |  |
| 1148 | gctctttcacccgtatcgctttaag |  |
