# Supplementary material for: Cis-by-Trans Regulatory Divergence Causes the Asymmetric Lethal Effects of an Ancestral Hybrid Incompatibility Gene
Source: PLoS Genet. 2012 Mar 22;8(3):e1002597. doi: 10.1371/journal.pgen.1002597 (PMC3310770; doi:10.1371/journal.pgen.1002597)
Supplement: Table S3 — A single dose of transgenic mel-Lhr suppresses hybrid rescue by D. simulans Lhr1. D. melanogaster females of the genotype y1,w67c23; P{w+mC = lacW}l(2)k01209[k08901a]/CyO; φ{mel-Lhr-HA}/+ were mated to D. simulans Lhr1 males. P{w+mC = lacW}l(2)k01209[k08901a] is abbreviated as Df(Lhr) and is described in Materials and Methods. Hybrid males were scored as follows: Df(Lhr)/Lhr1 progeny are Cy+ and have straight wings; Bal/Lhr1 progeny carry the CyO balancer chromosome and have Cy (curly) wings. The attP2 site into which φ{mel-Lhr-HA} is integrated is marked with the yellow+ body-color gene. Hybrid male progeny that inherit the transgene are therefore wild type for body color, while the sibling brothers are yellow bodied. Hybrid female progeny with and without the transgene cannot be distinguished because they inherit the X chromosome from D. simulans Lhr1 fathers that is wild type for the yellow locus. RT = room temperature. (DOCX) [file pgen.1002597.s007.docx]

Table S3: A single dose of transgenic *mel-Lhr* suppresses hybrid rescue by *D. simulans Lhr^1^*.

|  | Number of hybrid females | | |  | Number of hybrid males | | | | |
| --- | --- | --- | --- | --- | --- | --- | --- | --- | --- |
|  | *Df(Lhr)/Lhr^1^* |  | *Bal/Lhr^1^* |  | *Df(Lhr)/Lhr^1^* | |  | *Bal/Lhr^1^* | |
| Temp. |  |  |  |  | *φ{Lhr} /+* | *+/+* |  | *φ{Lhr} /+* | *+/+* |
| 18°C | 124 |  | 173 |  | 0 | 67 |  | 0 | 62 |
| RT | 198 |  | 149 |  | 0 | 68 |  | 0 | 52 |
